# Undifferentiated carcinoma with osteoclast-like giant cells of the pancreas harboring *KRAS* and *BRCA* mutations: case report and whole exome sequencing analysis

**DOI:** 10.1186/s12876-020-01351-7

**Published:** 2020-06-26

**Authors:** Guangjian Yang, Jiangxia Yin, Kai Ou, Qiang Du, Wenhao Ren, Yujing Jin, Liming Peng, Lin Yang

**Affiliations:** 1 Department of Medical Oncology, National Cancer Center/National Clinical Research Center for Cancer/ Cancer Hospital, Chinese Academy of Medical Sciences and Peking Union Medical College, Beijing, 100021 China; 2 Department of Oncology, Shouguang Hospital of Traditional Chinese Medicine, Weifang, 262700 China; 3 Department of Pathology, National Cancer Center/National Clinical Research Center for Cancer/ Cancer Hospital, Chinese Academy of Medical Sciences and Peking Union Medical College, Beijing, 100021 China; 4 Department of Medical Imaging, National Cancer Center/National Clinical Research Center for Cancer/ Cancer Hospital, Chinese Academy of Medical Sciences and Peking Union Medical College, Beijing, 100021 China

**Keywords:** Undifferentiated carcinoma with osteoclast-like giant cells, Pancreatic ductal adenocarcinoma, Pancreas, Case report, Whole exome sequencing

## Abstract

**Background:**

Undifferentiated carcinoma with osteoclast-like giant cells (UC-OGC) is an extremely uncommon pancreatic neoplasm that comprises less than 1% of all exocrine pancreatic tumors. To date, cases and data from whole-exome sequencing (WES) analysis have been reported by specific studies. We report a case of pancreatic UC-OGC with a literature review, and provide novel insights into the molecular characteristics of this tumor entity.

**Case presentation:**

A 31-year-old male presented with intermittent abdominal pain for several months, and positron emission tomography (PET) showed isolated high metabolic nodules during the pancreatic uncinate process that were likely to be malignant disease. Pathological examination after radical excision revealed UC-OGC associated with poorly differentiated adenocarcinoma at the head of the pancreas. The disease recurred 7.4 months after radical surgery. The *KRAS* p.G12D (c.35G > A) and somatic *BRCA2* p.R2896C (c.8686C > T) mutations were detected by subsequent WES analysis. The patient showed no response to platinum-based systemic chemotherapy, and his condition quickly worsened. He finally died, with an overall survival of 1 year.

**Conclusions:**

As an extremely uncommon tumor entity, UC-OGC is really a unique variant of conventional pancreatic ductal adenocarcinoma due to its similarities, as shown by genomic WES analysis. Clinical examination and molecular analysis by WES could further indicate potential treatment strategies for UC-OGC.

## Background

Pancreatic cancer is the thirteenth most malignancy worldwide [1], with a high mortality that is equal to the incidence. Pancreatic ductal adenocarcinoma (PDAC), as the most common pathologic type, is associated with poor treatment response and poor prognosis. The reported evidence has revealed that the molecular characteristics of PDAC include alterations in the driver gene *KRAS* and the tumor suppressor genes *TP53*, *CDKN2A* and *SMAD4* [2–6]. Undifferentiated carcinoma with osteoclast-like giant cells (UC-OGC), as a variant of anaplastic carcinoma of the pancreas, is observed extremely rarely in clinical practice [7, 8]. Worldwide, sporadic case reports have indicated that UC-OGC comprises less than 1% of all exocrine pancreatic tumors [9, 10]. Analysis of 38 UC-OGC cases demonstrated that it showed a better clinical course compared with that of conventional PDAC [8]. In addition, a few molecular studies of UC-OGC reported that *KRAS* mutations most frequently occurred, which was similar to that observed in PDAC [11–13]. Additionally, one detailed study reported the molecular features of UC-OGC by performing whole-exome sequencing (WES) analysis [14], and all these results implied that pancreatic UC-OGC was analogous to PDAC. To date, more cohorts of patients are needed to investigate the pathological and genetic features of this unique tumor variant. Herein, we report a case of pancreatic UC-OGC harboring the *KRAS* p.G12D mutation and somatic *BRCA2* mutation, as detected by WES, in a patient experienced reduced disease-free survival (DFS) and overall survival (OS). Furthermore, we provide a literature review of UC-OGC studies and analyze them to obtain novel insights regarding the molecular characteristics of this tumor entity.

## Case presentation

**Fig. 1:**
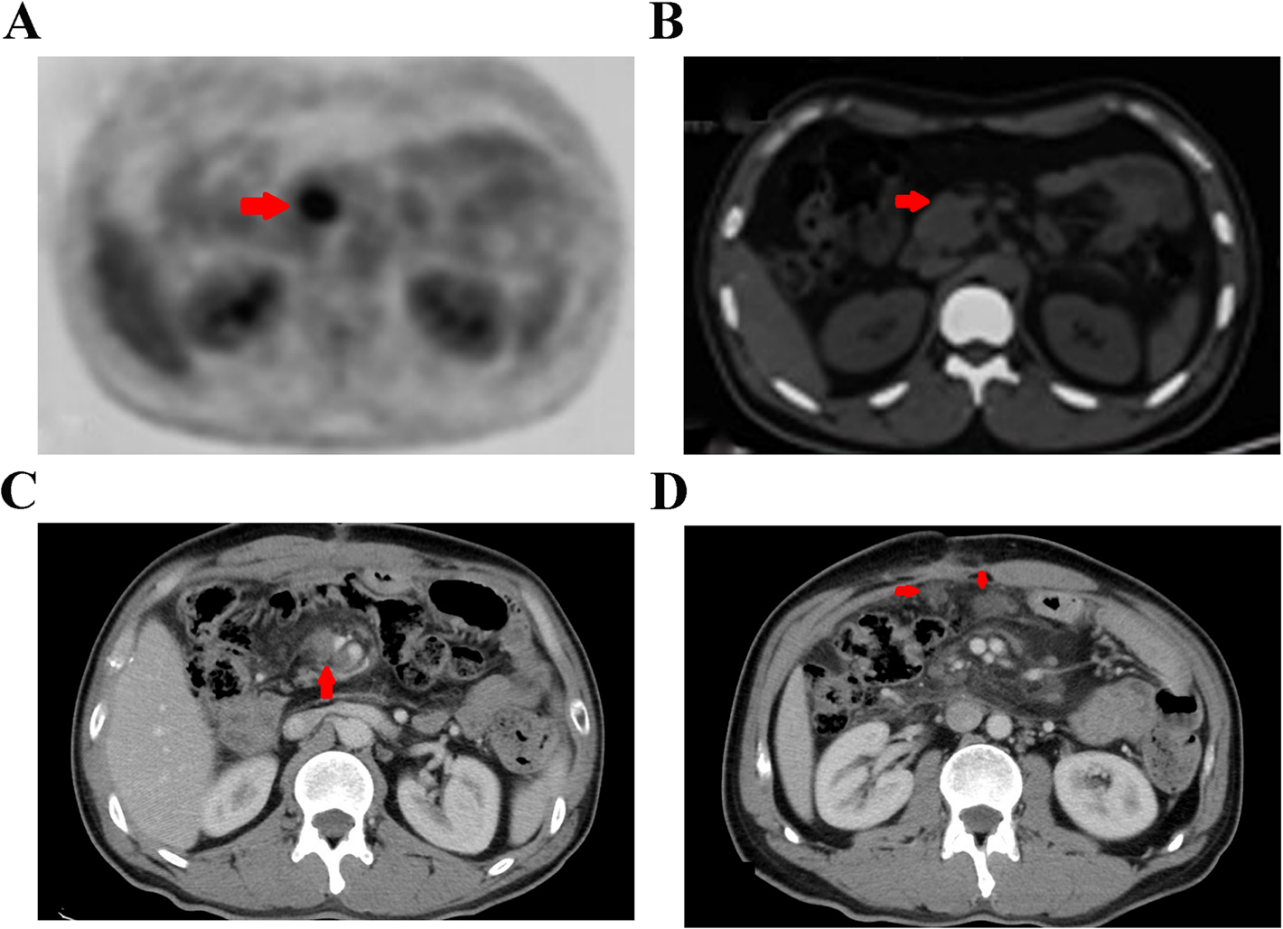
The PET showed high metabolic nodules at pancreatic uncinated process and inclined to be malignant disease at baseline (**a**, **b**). The contrasted CT scan showed multiple lymphatic metastases in the mesenteric region(**c**) and peritoneum (**d**) beyond termination of adjuvant chemotherapy

A 31-year-old male with no past medical or family history of disease presented with intermittent abdominal pain lasting almost 2 months, and he was admitted to the local hospital on February 28, 2017. Positron emission tomography (PET) showed isolated high metabolic nodules during the pancreatic uncinate process that were likely to represent malignant disease (Fig.1a, b).

**Fig. 2:**
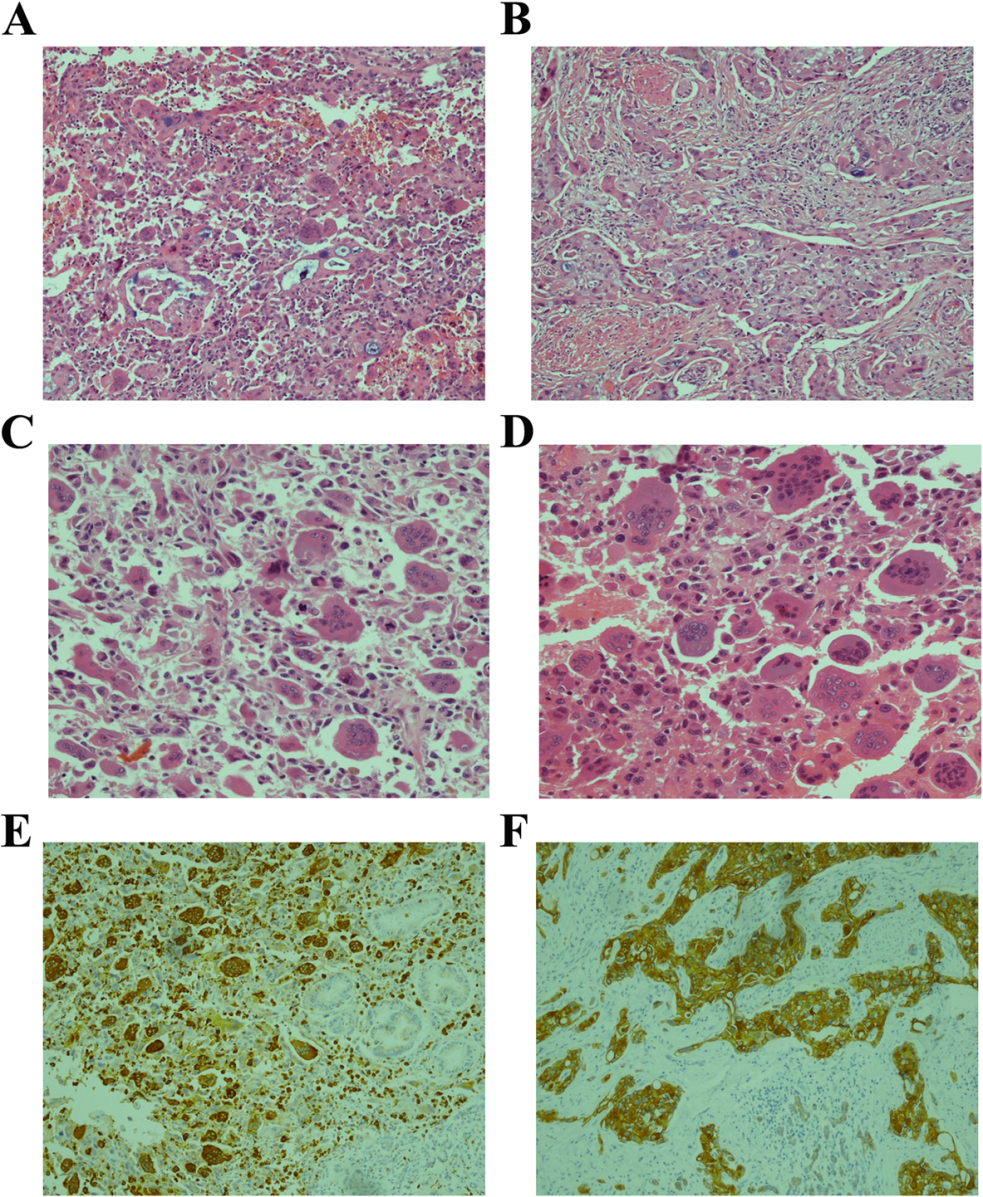
Histological features of pancreatic undifferentiated carcinoma with osteoclast-like giant cells (UC-OGC) under 100X (H&E, **a**). UC-OGC associated with poorly differentiated ductal adenocarcinoma component under 100X (H&E, **b**). The UC-OGC composed of anaplastic carcinoma and intermixed with pleomorphic neoplastic mononuclear cells and multinucleated osteoclast-like giant cells under 200X (H&E, **c** and **d**). Osteoclast-like giant cells of the tumor were stained positive for CD68 (IHC, **e**). Staining was positive for CK7 diffusely in the PDAC component of the tumor (IHC, **f**)

The patient then underwent radical pancreaticoduodenectomy on March 9, 2017. Pathological examination after radical excision showed poorly differentiated ductal adenocarcinoma associated with UC-OGC at the head of the pancreas (Fig.2a-d). Immunohistochemistry staining revealed that the cells were positive for CD68 and CK7, whereas the cells were negative for vimentin and S-100 (Fig. 2e, f). The tumor was measured to be 3 × 3 × 2 cm in size and exhibited invasion of the nerves, nearby pancreatic tissues, duodenum and the lower part of the common bile duct. The surgical margins were negative, and there was no discovery of lymph node metastasis. The surgical-pathological staging of the tumor was IIA (T3N0M0) according to the 7th edition of the American Joint Committee on Cancer (AJCC)/Union for International Cancer Control (UICC) TNM staging system.

Adjuvant chemotherapy with gemcitabine and albumin-bound paclitaxel was administered starting on April 10, 2017 for six cycles, and the toxicity was acceptable. However, the patient developed a backache 2 months after the termination of adjuvant chemotherapy. The contrasted computed tomography (CT) scan performed on November 27, 2017 showed multiple lymph node metastases in the mesenteric region (Fig. 1c) and peritoneum (Fig. 1d) with a serum CA199 level > 900 U/ml. Exploratory laparotomy was performed on November 29, 2017, and affirmed peritoneal metastasis was confirmed by peritoneal biopsy. The patient afterwards received systemic chemotherapy with the FOLFIRINOX regimen (combination of oxaliplatin, irinotecan, fluorouracil and leucovorin) for two cycles. Unfortunately, the serum tumor marker CA199 level was elevated to 1595 U/ml after two treatment cycles, and the patient’s condition deteriorated due to obvious myelosuppression and digestive tract toxicity caused by the chemotherapeutic drugs. Finally, he had to suspend chemotherapy and was admitted to our hospital on January 11, 2018.

WES analysis was performed, and the *KRAS* p. G12D (c. 35G > A) and somatic *BRCA2* p. R2896C (c. 8686C > T) mutations were detected in both surgical formalin-fixed paraffin-embedded (FFPE) tumor tissues and plasma ctDNA samples. Additionally, WES indicated that the tumor did not show microsatellite instability (MSI) and did not present a high tumor mutational burden (TMB). Considering the poor condition of the patient and the fact that the polyadenosine diphosphate-ribose polymerase (PARP) inhibitor olaparib was not available, we administered apatinib combined with tegafur/gimeracil/oteracil potassium capsules (S-1) for his disease. However, the patient’s condition worsened rapidly with the occurrence of fever, jaundice and vomiting after 1 month of treatment with this regimen, and eventually he died on March 12, 2018. The disease-free survival (DFS), which was defined as the time from radical surgery to disease recurrence, was just 7.4 months. The overall survival (OS), which was defined as the time between the primary diagnosis of UC-OGC and death, was only 12.6 months.

## Discussion and conclusion

Undifferentiated carcinoma of the pancreas, is a highly malignant tumor that tends to exhibit invasion of the perineum, lymph nodes and blood vessels and is called “giant cell carcinoma” or “pleomorphic large cell carcinoma” [15]. Tumors with osteoclast-like giant cells (OGCs) have been documented in a variety of organs, including the kidney, breast, thyroid gland, heart, parotid gland and skin [7, 16–18]. The UC-OGC is composed of pleomorphic neoplastic mononuclear cells that are and intermixed with large non-neoplastic multinucleated giant cells, as observed under microscopy [19], and it is suggested that UC-OGC is derived from epithelial tumors and the components of vimentin-positive carcinoma, which represent the mesenchymal transition of ductal cells [20, 21]. Based on the pathological features, the World Health Organization (WHO) had classified UC-OGC as a unique PDAC variant in 2010 [22].

The OGCs within the background of anaplastic malignant cells in UC-OGC are commonly considered to be of benign histiocytic origin, which has been supported in several cases by their immunoreactivity with CD68 [16]. Currently, it is hypothesized that OGC recruitment is a result of chemotactic factors produced by neoplastic cells and is indicative of a better prognosis [16]. Notably, such tumors can be classified as pure UC-OGC if they are not associated with a distinct neoplasm with a different morphology [14]. Luchini et al. [14] reported that the median OS (mOS) of 16 analyzed UC-OGC patients was 20 months, and the mOS of patients with pure UC-OGC was significantly higher than that of patients with associated PDAC (36 vs. 15 months, *P* = 0.04). Furthermore, it revealed an UC-OGC associated with PDAC conferred a five-fold increased risk of death [14], which was in accordance with the survival data reported by Muraki et al. [8]. The presence of UC-OGC in our case was confirmed by CD68 staining in the margin of undifferentiated tumors, and immunoreactivity with CK7 showed the presence of an associated adenocarcinoma component, which proved that this particular case was not pure UC-OGC. The 31-year-old male patient in our case survived for only 1 year, which was similar to the length of survival previously reported above [8, 14].

WES analysis of 8 UC-OGC patients had revealed that *KRAS* oncogenic mutations were identified in all analyzed cases, which implied that this tumor entity shared similar genomic features with conventional PDAC [14]. In addition, other previous studies also indicated the prevalence of *KRAS* mutations in UC-OGC [11–13, 23]. Based on the WES outcome for the UC-OGC cohort reported by Luchini et al. [14], all variants of *KRAS* mutations were found in codon 12, including the G12V, G12D and G12R mutations. In addition, additional somatic mutations in the tumor suppressor genes *TP53*, *CDKN2A* and *SMAD4* were detected in these UC-OGC cases, which further indicated that UC-OGC is a unique phenotype of PDAC due to the fact that these alterations either commonly appear in PDAC [14]. Additionally, Luchini et al. found the same *SERPINA3* variant (p.M290L) in a hotspot region in two UC-OGC cases and suggested that it may be an oncogene that had been previously reported in squamous cell carcinoma in the cervix [14]. *SERPINA3* encodes α-1-antichymotrypsin, which inhibits a plasma protease belonging to the serine protease inhibitor class [24]. Of note, the upregulation of *SERPINA3* is correlated with increases in cancer cell migration and invasion, and indicated a poor prognosis for several cancer types [25, 26]. WES analysis also suggested that *GLI3* was a driver gene of UC-OGC, as it was detected in two cases [14]. *GLI3*, as a target of microRNAs and transcription factors of the Hedgehog signalling pathway, is known to be upregulated in multiple cancers, in which it results in cancerous cell behaviour such as anchorage-independent growth, angiogenesis, proliferation and migration [27]. Except for the above mutations, it was difficult to interpret the importance of the other nonsynonymous mutations in *MEGF8*, *MAGEB4* and *TTN* detected by WES [14]. Muller et al. reported that the dosage gain in *KRAS* p. G12D dosage gain was not only related to early tumor progression, but also associated with metastasis in PDAC [28]. Unfortunately, there is currently no highly selective agent to suppress *KRAS*-mutated cancer. The WES analysis of our case indicated that the *KRAS* p. G12D mutation functioned as a major driver that resulted in the activation of downstream signalling pathways and high-grade disease malignancy. The patient suffered a pancreatic tumor at a young age and his disease progressed rapidly within an extremely short time after the previous radical operation. These results indicated that *KRAS* mutations in both in UC-OGC and PDAC result in the activation of oncogenes, which results in a poor prognosis, and that targeted agents against *KRAS* oncogenic mutations are urgently needed.

PDAC has been reported to have an immunosuppressive tumor microenvironment with a high programmed cell death-ligand 1 (PD-L1) expression, and in turn, the overexpression of PD-L1 inhibited the cytotoxic effects of activated T-cells [29]. Several studies have indicated that all indicated PD-L1 expression in PDAC is associated with a significantly poorer prognosis compared to that in patients without PD-L1 expression [29–34]. Luchini et al. investigated the PD-L1 expression patterns in pancreatic UC-OGC and finally found that PD-L1 was more frequently expressed in cases associated with PDAC than in cases associated with pure UC-OGC (*P* = 0.04), and PD-L1-positive UG-OGC was associated with a three-fold (*P* = 0.034) higher risk of mortality than PD-L1-negative UC-OGC [35]. In addition, the mismatch repair (MMR) system plays a crucial role in the repair of DNA sequence mismatches during replication. Defects in the MMR system (dMMR) could lead to errors in DNA replication, resulting in a high-TMB or increased MSI [36]. Thus, a high neoantigen load that increases proinflammatory cytokine levels and the activation of T cells is accumulated due to somatic mutations and contributes to the immunogenicity of MSI tumors with a sensitivity to immune checkpoint blockade [37]. Nevertheless, the prevalence of MSI/dMMR in PDAC is likely to be much lower than that in other gastrointestinal cancers, with only a 0–0.8% prevalence rate, as previously reported [38, 39]. Salem et al. analyzed 870 PDAC cases and found a low prevalence (1.4%) of high TMB in PDAC, and the majority of cases had a low TMB in either MSI-high or MSI-low patients [40]. A genomic profile analysis with a large sample size including 3594 PDAC cases [6] demonstrated that MSI-high and/or TMB-high status was detected in only 0.5% of samples [6]. In addition, *KRAS*, *TP53*, *CDKN2A* and *SMAD4* were the most frequently altered genes, and *KRAS* mutations ranked the first, with a prevalence of 88%. Additionally, alterations of the *BRCA* and *FANC* genes, which encode DNA damage repair proteins, were found in 14% of PDAC cases [6]. The tumor did not show MSI and did not present a high-TMB in our case, and the PD-L1 expression of this case was unknown. Based on the description given above, the patient associated with our case had no indication for immunotherapy.

In addition to the common *KRAS* oncogenic mutations, additional somatic *BRCA2* alterations were detected by WES in this case. Pancreatic cancer was reported to be the third most common cancer associated with *BRCA* mutations [41]. Approximately 7% of patients with pancreatic cancer carried germline mutations in *BRCA1/2*, and the frequency of *BRCA1/2* mutation carriers was estimated to be at 4.9 to 26% in familial pancreatic cancer [42]. To date, the largest reported PDAC case series involving patients with germline *BRCA* mutations showed that the median OS was 27.6 months [43]. Ashkenazi Jews have been the population with the highest prevalence of BRCA1/2 mutations in pancreatic cancer, with approximately 96% of patients having mutations in BRCA1/2 (BRCA1 185delAG, BRCA1 5382insC, or BRCA2 6174delT), and the *BRCA2* 6174delT variant is the most common variant in familial pancreatic cancer [44]. The PARP inhibitor olaparib had an objective response rate (ORR) of 21.7% in heavily pretreated pancreatic cancer patients with germline *BRCA1/2* mutations in a phase II study [45]. A randomized phase III study [46] showed that after first-line platinum-based chemotherapy, olaparib functioned as a maintenance therapy in pancreatic cancer patients with germline *BRCA1/2* mutations and significantly prolonged the median PFS compared with that in patients subjected to maintenance with a placebo (7.4 vs. 3.8 months, *P* = 0.004).

Advances in pancreatic cancer are lacking, as it is actually a highly heterogeneous disease resistant to conventional cytotoxic chemotherapeutic drugs or targeted agents [47]. The chemotherapy regimen of FOLFIRINOX (combination of oxaliplatin, irinotecan, fluorouracil and leucovorin) [48] or gemcitabine plus albumin-bound paclitaxel [49] is the preferred first-line recommendation for the treatment of in metastatic PDAC. Some evidence has also shown that *BRCA*-deficient cells are more susceptible to platinum than *BRCA*-proficient cells [50, 51], which has been supported by several clinical trials [52, 53]. The new version of the National Comprehensive Cancer Network (NCCN) Guidelines had recommended gemcitabine/cisplatin chemotherapy as one of the first-line regimens for *BRCA1*/*BRCA2*-mutated PDAC [54]. Waddell et al. reported that 4 patients with unstable genomes or a high *BRCA* mutational signature burden had robust complete or partial responses to platinum-based chemotherapy among 8 PDAC patients who received the same regimen, while 3 patients without these characteristics did not respond. Subsequent research also indicated that *BRCA2*-mutant patient-derived xenografts (PDXs) responded to cisplatin, and PDXs without mutations in a *BRCA* pathway gene failed to respond to cisplatin as well [55]. All these findings demonstrated that mutations in *BRCA* pathway genes or genomic instability had potential implications for the selection of PDAC treatment. In our case, the patient was a carrier of the somatic *BRCA2* mutant (p. R2896C), which has not been characterized to have known functional consequences. Subsequent bioinformatics analysis with various prediction software packages predicted the *BRCA2* p. R2896C mutation to be neutral. The disease in this patient rapidly progressed after only two cycles of platinum-based chemotherapy, and treatment with a PARP inhibitor was not possible owing to the presence of a non-germline *BRCA2* mutation.

Based on the mutational landscape of the genomics by WES, Waddell et al. [55] classified PDAC into four subtypes based on potential clinical utility according to exome and copy number variation (CNV) analyses including stable, locally rearranged, scattered and unstable. In the stable subtype, tumor genomes showed evidence of ≤50 structural variations that were located randomly throughout the genome. The locally rearranged type, it exhibited at least 50 focal variations on one or two chromosomes and nearly 1/3 the tumors of this subtype contained regions of copy number gain that harbored certain oncogenes. The scattered subtype exhibited nonrandom chromosomal damage and fewer than 200 structural variations. The unstable subtype exhibited a large number of structural variations (> 200), and the high level of genomic instability suggested defects in DNA maintenance and potentially showed sensitivity to DNA-damaging agents. In addition, Bailey et al. defined pancreatic cancer according to another four subtypes, including squamous, pancreatic progenitor, immunogenic and aberrantly differentiated endocrine exocrine [5]. These different types are associated with distinct histopathological characteristics, and each inferred the presence of different mechanisms of the molecular evolution of pancreatic cancer. To some degree, the assessment of the subtype can guide accurate therapeutic selection for pancreatic cancer. Furthermore, researchers have identified five new susceptibility loci for pancreatic cancer in the Chinese population to provide effective markers for the early screening and diagnosis of this very malignant cancer [56]. In this case, WES analysis revealed that the CNV in the *SOX9* gene gained approximately 1.11% variarion, whereas the CNV results for the *KRAS* and *BRCA2* genes were normal. Based on the mutational landscape of pancreatic cancer illustrated above, the case in this study deserved to be classified as the stable subtype owing to the presence of less than 50 structural variation events in the CNV.

In conclusion, although pancreatic UC-OGC is extremely uncommon and complex, the current evidence has clarified that it is a unique variant of conventional PDAC due to the genomic similarities between it and PDAC revealed by WES analysis. Assessment of the clinical and molecular characteristics by WES would further provide potential treatment strategies for this tumor entity.

## Data Availability

To protect the patient’s privacy, the data for the current case were not allowed to be shared, which was requested by the patient in the written application.

## Abbreviations

AJCC/UICC: American Joint Committee on Cancer/Union for International Cancer Control
CNV: Copy number variation
CT: Computed tomography
DFS: Disease-free survival
dMMR: Defects in mismatch repair system
MMR: Mismatch repair
mOS: Median overall survival
MSI: Microsatellite instability
NCCN: National Comprehensive Cancer Network
ORR: Objective response rate
OS: Overall survival
PARP: Poly adenosine diphosphate-ribose polymerase
PDAC: Pancreatic ductal adenocarcinoma
PD-L1: Programmed cell death-ligand 1
PDX: Patient-derived xenografts
PET: Positron emission tomography
S-1: Tegafur gimeracil oteracil potassium capsules
TMB: Tumor mutational burden
UC-OGC: Undifferentiated carcinoma with osteoclast-like giant cells
WES: Whole-exome sequencing
WHO: World Health Organization
